# Genome-wide association and genomic prediction for host response to porcine reproductive and respiratory syndrome virus infection

**DOI:** 10.1186/1297-9686-46-18

**Published:** 2014-03-04

**Authors:** Nicholas J Boddicker, Angelica Bjorkquist, Raymond RR Rowland, Joan K Lunney, James M Reecy, Jack CM Dekkers

**Affiliations:** 1 Department of Animal Science, Iowa State University, Ames, Iowa 50011, USA; 2 College of Veterinary Medicine, Kansas State University, Manhattan, Kansas 66506, USA; 3 United State Department of Agriculture, Agricultural Research Services, Beltsville Agricultural Research Center, Beltsville, Maryland 20705, USA

## Abstract

**Background:**

Host genetics has been shown to play a role in porcine reproductive and respiratory syndrome (PRRS), which is the most economically important disease in the swine industry. A region on *Sus scrofa* chromosome (SSC) 4 has been previously reported to have a strong association with serum viremia and weight gain in pigs experimentally infected with the PRRS virus (PRRSV). The objective here was to identify haplotypes associated with the favorable phenotype, investigate additional genomic regions associated with host response to PRRSV, and to determine the predictive ability of genomic estimated breeding values (GEBV) based on the SSC4 region and based on the rest of the genome. Phenotypic data and 60 K SNP genotypes from eight trials of ~200 pigs from different commercial crosses were used to address these objectives.

**Results:**

Across the eight trials, heritability estimates were 0.44 and 0.29 for viral load (VL, area under the curve of log-transformed serum viremia from 0 to 21 days post infection) and weight gain to 42 days post infection (WG), respectively. Genomic regions associated with VL were identified on chromosomes 4, X, and 1. Genomic regions associated with WG were identified on chromosomes 4, 5, and 7. Apart from the SSC4 region, the regions associated with these two traits each explained less than 3% of the genetic variance. Due to the strong linkage disequilibrium in the SSC4 region, only 19 unique haplotypes were identified across all populations, of which four were associated with the favorable phenotype. Through cross-validation, accuracies of EBV based on the SSC4 region were high (0.55), while the rest of the genome had little predictive ability across populations (0.09).

**Conclusions:**

Traits associated with response to PRRSV infection in growing pigs are largely controlled by genomic regions with relatively small effects, with the exception of SSC4. Accuracies of EBV based on the SSC4 region were high compared to the rest of the genome. These results show that selection for the SSC4 region could potentially reduce the effects of PRRS in growing pigs, ultimately reducing the economic impact of this disease.

## Background

For decades the swine industry has been battling the economically important disease of porcine reproductive and respiratory syndrome (PRRS), caused by the PRRS virus (PRRSV) but success has been limited. Since the infection mechanisms of the virus are not fully understood, vaccine development is a challenge [1]. There has been no genetic selection of pigs for PRRS resistance or tolerance due to the lack of good DNA markers for marker-assisted selection and lack of a good indicator trait for indirect selection. Recently, a quantitative trait locus (QTL) was identified on *Sus scrofa* (SSC) 4 that explains a considerable amount of the total genetic variance for serum viremia and weight gain of weaned piglets following experimental infection [2]. In subsequent work, the effect of this ~1 Mb region was identified in three unrelated populations, encompassing five experimental challenge trials of 200 weaned pigs [3]. Many of the single nucleotide polymorphisms (SNPs) in this ~1 Mb region are in very high linkage disequilibrium, which makes identification of the causative mutation difficult; a single tag SNP (SNP WUR10000125) appears to track the effect of the region with the B allele being associated with PRRS tolerance [2,3]. The fore mentioned studies did not thoroughly investigate the rest of the genome, which may contain pertinent information for host response to PRRSV infection. The addition of three trials to the data analyzed in Boddicker et al. [3] has potentially increased the power in order to find these, likely smaller, effects.

The objectives of the current study were to (1) re-estimate genetic parameters for host response to experimental PRRSV infection in growing pigs by including three additional, unrelated populations, (2) investigate additional genomic regions associated with weight gain and viremia in response to PRRSV challenge, (3) determine if there is a smaller informative haplotype block within the ~1 Mb region on SSC4 associated with host response to PRRSV infection, (4) determine which haplotypes within the SSC4 region are associated with the favorable effects of the QTL for viremia and weight gain, and (5) investigate accuracies of genomic estimated breeding values (GEBV) for host response to PRRS based on the SSC4 region and the rest of the genome, by using cross-validation.

## Methods

The Kansas State University Institutional Animal Care and Use Committee approved all experimental protocols for this study.

### Study design

A detailed description of the design, data collection, and molecular techniques used in the PRRS host genetic consortium trials has been previously published [4]. Briefly, each challenge trial of ~200 commercial pigs involved transporting animals at weaning (18–28 days of age) to Kansas State University, where they were subjected to a PRRSV challenge. Within a trial, pigs were from the same high health farm, except for trials 5 and 8, which each included pigs from two farms. All farms were free of PRRSV, *Mycoplasma hyopneumoniae*, and swine influenza virus. Upon arrival, pigs were randomly placed into pens of 10 to 15 pigs. After a 7-day acclimation period, pigs between 25 and 35 days of age (day 0), were experimentally infected intramuscularly and intranasally with 10^5^ (TCID50) of NVSL 97–7985, a highly virulent PRRSV isolate [5]. Blood samples were collected at -6, 0, 4, 7, 11, 14, 21, 28, 35, and 42 days post-infection (dpi). Body weight was measured at 0, 7, 14, 21, 28, 35, and 42 dpi. Pigs were euthanized at 42 dpi. Trials 7 and 8 were stopped at 35 dpi due to facility availability.

Viremia was measured using a semi-quantitative TaqMan PCR assay for PRRSV RNA, as described in Boddicker et al. [2]. Assay results were reported as the log_10_ of PRRSV RNA copies per mL of serum. Ear tissue was collected from all pigs for DNA isolation. Tissue or genomic DNA from the sires of pigs in trials 1 through 3 and from available sires and dams for trials 4 through 8 was supplied by the breeding companies. Tissues or DNA samples were sent to GeneSeek Inc. (Lincoln, Nebraska) for genotyping with Illumina’s Porcine SNP60 BeadChip (San Diego, California).

Data from eight trials of up to 200 pigs were analyzed (Table 1). Trials 1 through 3 were described in Boddicker et al. [2] and included pigs of the same cross from a single breeding company. Trials 4 and 5 were described in Boddicker et al. [3] and included two unrelated populations from different breeding companies. Trials 6 through 8 are unique to this paper and were sourced from three additional breeding companies, with pigs that were unrelated to those in Boddicker et al. [2,3]. Pigs in trial 7 were sourced from the same breeding company as those in trial 4 but were produced using different sire and dam lines.

**Table 1:** Population structure of trials 1 through 8

				**WUR**	**Number offspring per family**			**Total number of offspring**			
**Trial**		**Breed**^**1**^	**N**	**frequency**^**4**^	**Min**	**Mean**	**Max**	**Barrows**	**Gilts**	**Total**	**Dead**^**5**^
1-3^2,3^	Sires	LR	33	0.22	1	17.1	114	565	0	565	48
	Dams	LW	204	0.08	1	2.8	6				
4	Sires	Duroc	6	0.08	8	32.5	50	109	86	195	2
	Dams	LW/LR/Y	33	0.10	1	5.9	13				
5	Sires	Duroc	10	0.12	2	19.9	42	109	90	199	14
	Dams	LR/Y	38	0.22	2	5.2	10				
6	Sires	LR	31	0.02	1	6.2	24	198	0	198	87
	Dams	LR	72	0.03	1	2.8	5				
7	Sires	Pietrain	6	0.42	20	32.8	40	109	88	197	3
	Dams	LW/LR/Y	28	0.20	1	7	13				
8	Sires	Duroc	14	0.11	2	12.4	34	97	101	198	16
	Dams	Y/LR	34	0.07	1	4.5	17				

^1^*LR* Landrace, *LW* Large White, *Y* Yorkshire; ^2^trials 1 through 3 consisted of the same cross from one breeding company; ^3^trials 1 and 2 were Landrace by Large White crosses; for trial 3, 121 piglets were from Landrace sires by Large White dams crosses and 63 piglets were from the reciprocal cross of Large White sires by Landrace dams; ^4^frequency of the favorable allele (B) at SNP WUR10000125 on SSC4; for trials 1–3, dam genotypes were not provided, so frequencies were determined using ordered genotypes of the offspring; ^5^dead prior to 42 days after infection.

Table 1 provides an overview of the population structure by trial. See Boddicker et al. [2] for further details on trials 1 through 3 and Boddicker et al. [3] for further detail on trials 4 and 5. Pigs in trial 6 were purebred Landrace (LR). Pigs in trial 7 were from Pietrain sires and Large White (LW)/LR/Yorkshire dams. Trial 8 pigs were from Duroc sires and Yorkshire/LR dams. Across all eight trials, 175 pigs died before 42 dpi (Table 1). Dead pigs were necropsied and subsequent gross and microscopic pathology by a board-certified pathologist identified PRRS associated disease as the major source of mortality, except for trial 6. Death loss was high in trial 6, 46% by day 42, due to secondary bacterial infections, as identified by pathology, including *Escherichia coli, Streptococcus suis, Staphylococcus aureus,* and *Mycoplasma hyopneumoniae.*

### Phenotypic traits and pedigree

Details on the phenotypic traits analyzed are in Boddicker et al. [2,3]. Briefly, VL was quantified as the area under the curve for log-transformed serum viremia at 0, 4, 7, 11, 14, and 21 dpi. Weight gain to 42 dpi (WG) was calculated as body weight (BW) at 42 dpi minus BW at day 0 dpi; for trials 7 and 8 WG35 was calculated. Mortality was defined as death prior to the end of the experiment. Edits for trials 1–3 are in Boddicker et al. [2] and in Boddicker et al. [3] for trials 4 and 5. Briefly, edits for VL removed 34 individuals from the first 3 trials and 15 for trials 4 and 5. For WG, 47 and 18 individuals were removed, respectively. Edits removed 88 individuals from trials 6 through 8, with 86 due to death prior to 21 dpi and two with missing viremia data. For WG42, 111 individuals were removed from trials 6 through 8, all due to death prior to 42 dpi. The total number of individuals available after edits is in Table 2.

**Table 2:** Trait means and estimates (±SE) of heritability and litter effects (proportions of phenotypic variance) for virus load and weight gain after infection obtained from single trait pedigree-based ASREML analyses

**Trait**^**1**^	**n**	**Mean**	**SD**^**2**^	**Heritability**	**Litter**	**Phenotypic correlation**	**Genetic correlation**
VL, units	1416	106.9	7.34	0.44 ± 0.13	0.09 ± 0.05		
						-0.29 ± 0.03	-0.46 ± 0.20
WG42, kg	1373	14.4	4.03	0.29 ± 0.11	0.12 ± 0.05		

^1^*VL* viral load calculated as area under the curve of log-transformed viremia between 0 and 21 dpi and *WG42* weight gain from 0 dpi to 42 dpi; ^2^standard deviation calculated as the square root of the sum of animal, litter, and residual variances from the joint ASREML analysis of all 8 trials.

Recorded pedigrees (sire and dam) were checked and corrected using SNP genotypes, as described in Boddicker et al. [3]. Briefly, parent-offsping mismatch frequencies were calculated as the number of SNPs for which the parent and offspring had opposing homozygous genotypes divided by the total number of polymorphic SNPs for which the parent and offspring were both homozygous. If a parent-offspring pair had a mismatch frequency of less than 2%, then the named parent was accepted. Otherwise, offspring genotypes were compared to all possible parents and the most likely parent was chosen. The original pedigrees provided by the breeding companies were mostly correct but a few full- and half-sib families were reassigned to different sires and dams.

### Statistical analyses

Heritabilities and variances due to litter were estimated based on validated and corrected pedigree relationships, as determined from SNP genotypes (see Boddicker et al. [3] for details), with a single-trait animal model using the software ASREML [6]. Sex and the interaction of trial and parity of the sow were included as fixed factors and pen within trial, animal, and litter as random effects. Piglets were born from parities 1 to 7. Parities 3 through 7 were combined into one parity class. The effect of farm of origin for trials 5 and 8 was not significant (p > 0.43) and was excluded from the analyses. The genetic correlation between traits was estimated using a bivariate animal model with the same fixed and random factors as used in the single-trait models.

### Genome-wide association analyses

Associations of SNP genotypes with phenotypes were analyzed by fitting all SNPs simultaneously using Bayesian genomic selection methods [7], as implemented in the software GenSel [8], using the following mixed model:

$$y=\mathrm{Xb}+\overset{k}{\underset{j}{\sum }}z_{j}\alpha_{j}\delta_{j}+\varepsilon$$

where ***y*** = vector of phenotypic observations, ***X*** = incidence matrix relating fixed factors to phenotypes, ***b*** = vector of fixed factors of sex, pen within trial, and the interaction of trial and parity class, ***z***_***j***_ = vector of the genotype covariate for SNP *j* (*j* = 1 to *k*) based on the number of B alleles using Illumina’s (San Diego, California) genotype calling (coded 0, 1, 2, or equal to the trial average for missing genotypes), *α*_*j*_ = allele substitution effect for SNP *j*, and *δ*_*j*_ = indicator for whether SNP *j* was included (*δ*_*j*_ = 1) or excluded (*δ*_*j*_ = 0) in the model for a given iteration of the Markov chain Monte Carlo (MCMC). Pen within trial was included as a fixed factor, as opposed to a random effect in ASREML, because the current version of GenSel does not allow additional random effects. A total of 50 000 iterations were run for each analysis, with the first 5000 iterations discarded as burn-in. The probability of *δ*_*i*_ = 0 was set equal to π = 0.99. The Bayesian model was implemented using method Bayes-B [7]. Genomic regions associated with traits were identified using 1 Mb, non-overlapping windows using build 10.2 of the swine genome (http://www.ncbi.nlm.nih.gov/nuccore?term=199 Sus%20scrofa%2C%20whole%20genome%20shotgun%20sequence, accessed November 1, 2011).

### Haplotype analyses

The QTL previously identified on SSC4 included 38 SNPs, two of which had 0 called genotypes [2]. The remaining 36 SNPs in the ~1 Mb region on SSC4 were analyzed to remove uninformative groups of SNPs, with the goal of reducing the size of the ~1 Mb region. Linkage disequilibrium (LD) between SNPs in the ~1 Mb region was determined using Haploview [9]. Using a univariate animal model in ASREML that included sex, the interaction of trial and parity of the sow, and genotype for SNP WUR10000125 as fixed factors and pen within trial, animal, and litter as random effects, each of the remaining 35 SNPs was fitted as an additional covariate, one at a time, to determine if the additional SNP accounted for effects not captured by SNP WUR10000125. The threshold for discarding a SNP from the region was p < 0.05.

Parental and offspring genotypes for the 36 informative SNPs in the ~1 Mb region on SSC4 were ordered using the PHASE software [10], separately for each trial. Haplotypes with high probabilities, as identified by PHASE software, were analyzed with MEGA5 software [11] to establish a phylogenetic tree. The neighbor-joining method, with the p-distance option, was used to create the tree. Haplotypes identified in the ~1 Mb region and for the reduced region were analyzed.

Using the results from the phylogenetic tree for the reduced region, haplotypes that carried the B allele at SNP WUR10000125 were allocated to two groups based on phylogenetic distance from one another. The main effect of haplotype group was fitted as a fixed class factor in the abovementioned ASREML analysis to determine which haplotypes were associated with the desirable phenotypes of VL and WG. Due to the apparent dominance effect of the B allele, an individual had to have at least one copy of a haplotype that carried the B allele to be placed in that group. If the two groups of haplotypes that carried the B allele were not significantly different from each other but were significantly different from the group of haplotypes that carried the A allele, both B haplotypes groups were assumed to carry the favorable allele of the causative mutation. A similar procedure was used for the A haplotypes to determine whether all A haplotypes were associated with an unfavorable phenotype.

### Cross-validation

Accuracy of genomic predictions across populations was evaluated by cross-validation, which involved training on one population and validating on another population. Populations were defined by trial, except for trials 1 through 3, which were considered as one population, since pigs included in those three trials were crossbreds from the same lines and breeding company. Each population was validated twice, using a ‘reduced’ and a ‘full’ training population (Table 3). The training populations included all populations except for the population that was being validated, with some exceptions. For the reduced training populations, only one of the first three trials was used. Trial 3 was used for this purpose because it had the highest estimates of heritability of the three trials for both VL and WG (0.45 and 0.50, respectively). For the full training populations, trials 1 through 3 were included when validating on trials 4 through 8. When validating on trials 1 and 2, trial 3 was included in the training population, which resulted in some of the animals in the validation population to be related to animals in the training population.

**Table 3:** Training and validation populations used for cross-validation of genomic prediction for virus load (VL) and weight gain (WG)

**Validation population**			**Reduced training population**			**Full training population**		
**Trial**	**N for VL**^**1**^	**N for WG**^**2**^	**Trials**	**N for VL**^**1**^	**N for WG**^**2**^	**Trials**	**N for VL**^**1**^	**N for WG**^**2**^
1	185	177	4, 5, 6, 7, 8	871	843	3, 4, 5, 6, 7, 8	1,059	1,025
2	178	169	4, 5, 6, 7, 8	865	831	3, 4, 5, 6, 7, 8	1,059	1,025
3	176	168	4, 5, 6, 7, 8	936	919	-	-	-
4	195	193	3, 5, 6, 7, 8	875	842	1, 2, 3, 5, 6, 7, 8	1,218	1,178
5	184	183	3, 4, 6, 7, 8	864	832	1, 2, 3, 4, 6, 7, 8	1,229	1,188
6	123	106	3, 4, 5, 7, 8	884	858	1, 2, 3, 4, 5, 7, 8	1,290	1,265
7	194	194	3, 4, 5, 6, 8	884	858	1, 2, 3, 4, 5, 6, 8	1,219	1,177
8	188	182	3, 4, 5, 6, 7	884	858	1, 2, 3, 4, 5, 6, 7	1,225	1,189

^1^*VL* virus load calculated as area under the curve of log-transformed viremia between 0 and 21 days post infection (dpi); ^2^*WG* weight gain from 0 dpi to 42 dpi.

To allow predictive ability of the rest of the genome, excluding the SSC4 region, to be assessed, estimates of allele substitution effects for the training population were acquired using the additive Bayesian model described above but with genotype at SNP WUR10000125 included as a fixed class effect to account for the additive and non-additive effects of the QTL in this region on VL and WG [2]. Method Bayes-CPi was used rather than Bayes-B because it assumes a homogenous variance across all SNPs, which appeared appropriate given the results of the GWAS, and estimates parameter π from the data [7]. The starting value of π was set to 0.99. The GEBV of individual *i* in the validation population for the rest of the genome (excluding the SSC4 region) was predicted as:

$$\mathrm{GEB}V_{i}=\overset{k}{\underset{j=1}{\sum }}\left(z_{\mathrm{ij}}{\hat{\alpha }}_{J}\right)$$

where *k* = number of SNPs included in the prediction (58 277 SNPs), *z*_*ij*_ = genotype covariate of SNP *j* for animal *i* (coded 0, 1, 2, or trial average for missing genotypes), and α^j = allele substitution effect estimate for SNP *j* based on analysis of the training population. To estimate the accuracy of predictions based on the SSC4 region alone, the estimates of the additive and dominance effects of SNP WUR10000125 from the GenSel analysis of the training population were used to estimate the genotypic value based on the observed genotype for SNP WUR10000125 of each individual in the validation population. Accuracy of prediction, defined as the correlation between the GEBV and the true BV, were estimated as the correlation between the GEBV and adjusted phenotypes in the validation population divided by the square root of heritability (Table 2) obtained when analyzing all eight trials together [3]. Here, adjusted phenotypes were phenotypes adjusted for estimates of fixed effects (sex, pen within trial, and the interaction of trial and parity class) within the validation population. Estimates of accuracies of GEBV across all eight trials were then obtained by correlating adjusted phenotypes with GEBV deviated from their respective trial means, and dividing by the square root of heritability.

## Results and discussion

### Genetic parameters

Estimates of heritability, litter effects, and phenotypic and genetic correlations for VL and WG are in Table 2. Heritabilities for VL and WG were moderate at 0.44 and 0.29, respectively, and were of similar magnitude as those reported by Boddicker et al. [3] using a subset (trials 1–5) of the data analyzed here. In comparison to the estimates from the first five trials, the proportion of variance explained by litter effects decreased for VL (0.11 to 0.09) and increased for WG42 (0.09 to 0.12). These estimates of heritability provide solid evidence that genetic improvement of host response to PRRSV infection is possible. Phenotypic and genetic correlations between VL and WG were moderate and negative.

### Further analysis of the SSC4 region

#### ***SSC4 effects in trials 6–8***

**Figure 1:**
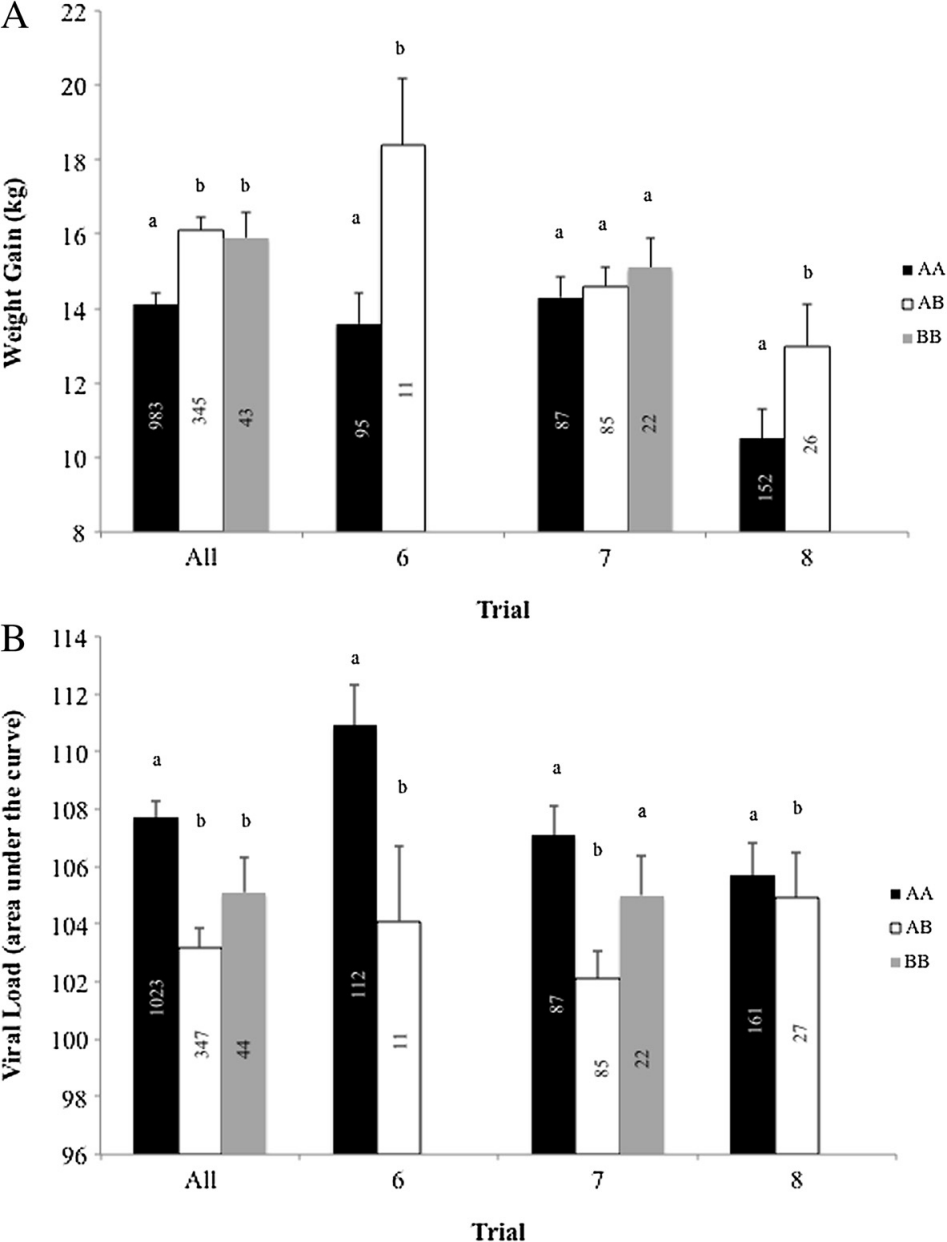
**Least squares means by genotype for SNP WUR10000125 across all trials 1 through 8 and within trials 6, 7, and 8 for weight gain from 0 to 42 days post infection (A) and viral load (B).** Viral load was calculated as area under the curve of log-transformed viremia from 0 to 21 days post infection. Within a trial, columns with a different letter are significantly different at p < 0.05. Numbers of individuals within genotype are listed in the bars.

Results of single-marker analyses for the most significant SNP in the SSC4 region (WUR10000125) that was identified by Boddicker et al. [2] for VL and WG42 for all trials combined, and trials 6 through 8 individually, are in Figure 1. Results for SNP WUR10000125 for trials 1 through 3 are in Boddicker et al. [2] and in Boddicker et al. [3] for trials 4 and 5. This SNP was significant for WG42 (Figure 1A) for trials 6 (p < 0.01) and 8 (p < 0.004), where susceptible AA animals gained 4.8 kg and 3.5 kg less, respectively, than AB animals. Although the effect was in the same direction for trial 7 for WG42, there was no statistical significance between the genotypes for this trial (p > 0.57). For VL (Figure 1B), the SNP was significant for trials 6 (p < 0.005) and 7 (p < 0.001) but not for trial 8 (p > 0.56). For trial 8, the effects of the SNP on VL were, however, in the same direction as for the other trials (i.e. increased VL for AA animals compared to AB animals). The non-significant effects of the WUR SNP on WG in trial 7 and on VL in trial 8 are probably false negatives because the effect of the SNP was significant for the other trait in each of these trials; it is less likely that the significant effects in trials 7 and 8 are false positives because the effect is present in the other trials for both traits [3]. These results show that the effect of this region was present in six unrelated populations from five different breeding companies.

#### ***Reducing the region on SSC4***

**Figure 2:**
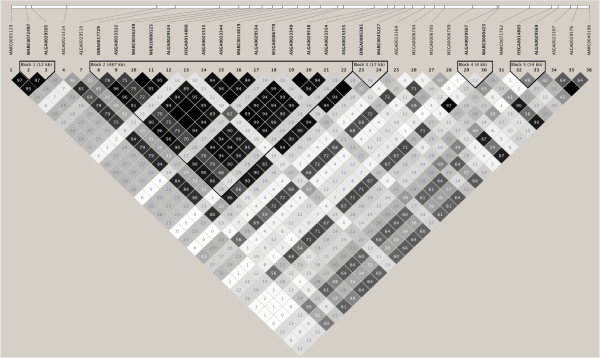
**Linkage disequilibrium (r**^**2**^**) plot of the 1 Mb region on *Sus scrofa *chromosome 4 across trials 1 through 8.** Black squares signify r^2^ = 100% and white squares signify r^2^ = 0%.

The region on SSC4 spans 1 284 081 base pairs and includes a total of 38 SNPs that are on the 60 k SNP panel, including two that are fixed and two for which no genotypes were called. Figure 2 shows a LD plot of the 34 polymorphic SNPs across trials 1 through 8. Haploview identified five haplotype blocks, including a block (Block 2) of 15 SNPs that spanned 487 kb that had very high LD amongst most SNPs. This block harbors SNP WUR10000125, which captured over 99% of the effects of the SSC4 region on VL and WG [2]. In general, LD is not expected to be the same between breeds and unrelated populations over such long distances. As an example, Amaral et al. [12] found very different LD patterns between a LW population, Ningxiang (a Chinese breed), and the European wild boar, across three different genomic regions. Differences in LD can result from mutation, recombination, selection, and drift. Block 2 is unique in the sense that LD was very high across six unrelated populations that consisted of different breeds (Figure 2).

Table 4 shows the p-value for the effects of each SNP in the SSC4 region on VL and WG42, when the effect of SNP WUR10000125 is accounted for in the model. The SNPs located before and after Block 2 all had p-values greater than 0.05 for both VL and WG42, indicating that these SNPs do not capture a significant amount of additional variation in VL or WG42 that is not already accounted for by SNP WUR10000125. Therefore, these SNPs are probably not in high LD with the causative mutation and were excluded from the candidate region. Many of the SNPs within Block 2 also had p-values greater than 0.05 but, with the high LD present and confounding among SNPs, Block 2 was not further reduced in size. Given these results, the ~1 Mb candidate region was reduced to a 487 kb region that probably harbors the causative mutation.

**Table 4:** **Statistical significance (p-values) of associations of single nucleotide polymorphisms within the 1 Mb region on chromosome 4 after accounting for the effects of SNP WUR10000125**^**1**^

			**LD (r**^**2**^**) with**	**P-values**	
**Number**	**SNP name**	**Position**	**WUR10000125**	**VL**^**2**^	**WG**^**3**^
1	MARC0005129	139136697	0.28	0.75	0.94
2	MARC0072497	139169386	0.27	0.68	0.7
3	ALGA0029505	139182285	0.27	0.52	0.95
4	ASGA0023314	139260674	0.79	0.62	0.10
5	MARC0027457	139409628	-	Fixed	
6	MARC0095662	139440014	-	Fixed	
7	ALGA0029510	139460508	0.35	0.18	0.76
**8**	**INRA0017729**	**139501559**	**0.96**	**0.52**	**< 0.01**
**9**	**ASGA0023322**	**139599067**	**0.75**	**0.65**	**0.7**
**10**	**MARC0056249**	**139642883**	**1.00**	**LD = 1**	
**11**	**WUR10000125**	**139666759**	**-**	**Used in model**	
**12**	**ALGA0029524**	**139694323**	**0.95**	**0.91**	**0.163**
**13**	**H3GA0014860**	**139717541**	**0.11**	**0.41**	**0.64**
**14**	**ASGA0023335**	**139739837**	**0.94**	**0.08**	**0.11**
**15**	**ASGA0023344**	**139772783**	**1.00**	**LD = 1**	
**16**	**MARC0014819**	**139800473**	**1.00**	**LD = 1**	
**17**	**ALGA0029534**	**139823807**	**0.94**	**0.08**	**0.11**
**18**	**M1GA0006779**	**139861417**	**0.17**	**0.39**	**0.92**
**19**	**ASGA0023349**	**139875297**	**0.94**	**0.03**	**0.39**
**20**	**ALGA0029538**	**139943624**	**0.94**	**0.03**	**0.37**
**21**	**ASGA0023354**	**139973857**	**0.90**	**0.41**	**0.81**
**22**	**ASGA0023355**	**139989537**	**0.19**	**0.17**	**0.57**
23	DRGA0005385	140011063	0.90	0.41	0.81
24	MARC0045227	140028977	0.16	0.97	0.91
25	ASGA0023369	140054732	0.04	0.62	0.72
26	M1GA0006784	140077330	0.72	0.61	0.79
27	M1GA0006785	140099804	0.04	0.97	0.96
28	M1GA0006789	140161976	0.12	0.71	0.73
29	ALGA0029567	140216349	0.08	0.07	0.82
30	MARC0000425	140220490	0.71	0.93	0.83
31	MARC0071762	140288666	0.19	0.06	0.4
32	H3GA0014885	140337996	0.15	0.98	0.96
33	ALGA0029569	140352057	0.30	0.64	0.74
34	ASGA0023397	140379843	0.64	0.81	0.94
35	ALGA0029576	140394822	0.34	0.89	0.29
36	MARC0040196	140420778	0.64	0.81	0.95

^1^The bolded area represents Block 2 of Figure 2; ^2^*VL* viral load calculated as area under the curve of log-transformed viremia between 0 and 21 days post-infection; ^3^*WG* weight gain from 0 dpi to 42 dpi.

#### ***Haplotype analysis***

**Figure 3:**
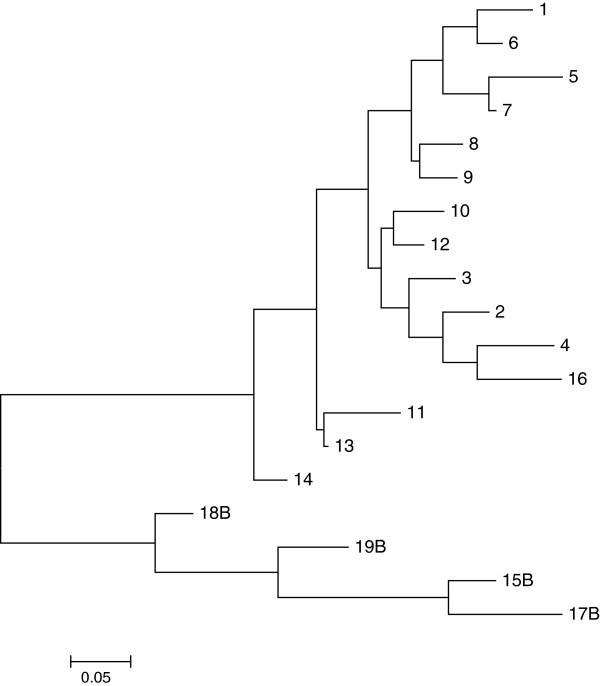
**Phylogenetic tree of the haplotypes present across trials 1 through 8 for the reduced 487 kb region on *Sus scrofa *chromosome 4.** The trees were created using the neighbor-joining, p-distance method in the Mega 5 software. Haplotypes with the B allele for SNP WUR10000125 are labeled with a B following the haplotype number.

A total of 77 unique haplotypes were identified in the original ~1 Mb region across all eight trials, 11 of which carried the B allele at SNP WUR10000125 (B haplotypes). After reducing the region to 487 kb, only 19 unique haplotypes remained, of which four carried the B allele. Table 5 shows the frequency of each B haplotype, along with the trials each haplotype was present in. Eight of the original eleven B haplotypes were combined into one haplotype (haplotype 17) after the region was reduced. This was the most common haplotype and it was present in all trials and in every parental breed. Therefore, it is probably the ancestral haplotype, with the remaining B haplotypes originating from recombination or mutation since the breeds were established.Figure 3 shows the phylogenetic tree for haplotypes in the 487 kb region, generated using Mega 5 software. Using only the unique haplotypes, the tree clearly grouped the haplotypes based on the allele present at SNP WUR10000125, with the B haplotypes grouped at the bottom of the tree in Figure 3.

**Table 5:** **Haplotypes for the 487 kb region (with arbitrary numbers corresponding to the phylogenetic tree in Figure **3**) that contain the B allele for SNP WUR10000125 on chromosome 4, along with the SNP sequence and the number of copies present in trials 1 through 8**

**Haplotype number**	**Haplotype SNP sequence**^**1**^	**Number of copies**	**Trials in which the haplotype was present**
17B	GAG**G**CGAGGGCCCAG	423	All
18B	GAG**G**CGCGGACAACG	21	5
19B	GAG**G**CGCGGACCCCG	1	1
15B	AGG**G**CGAGGGCCCAG	8	6

^1^Letter in bold is SNP WUR10000125.

**Figure 4:**
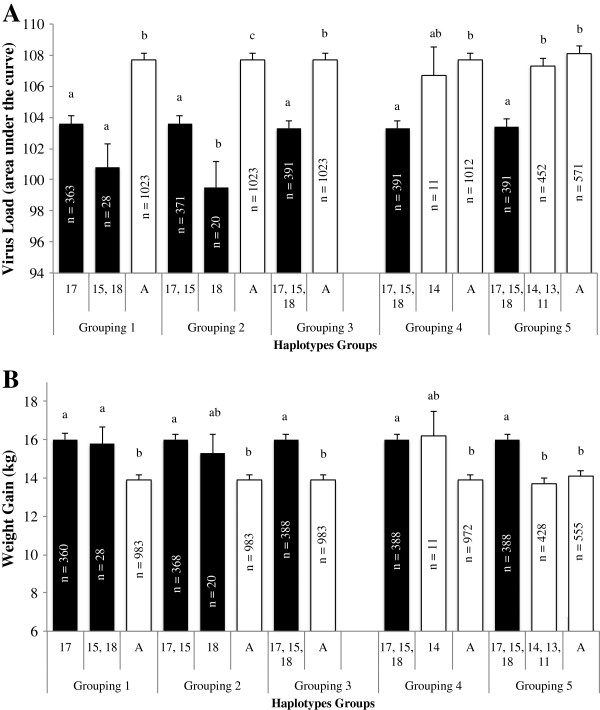
**Least squares means by haplotype group within a grouping based on cutting the phylogenetic tree for virual load (A) and weight gain (B).** Haplotype numbers refer to those identified in Figure 3; “A” refers to all A haplotypes. Within a grouping, columns with a different letter are significantly different at p < 0.05. Numbers of individuals within each haplotype group are listed in the bars. Black bars represent haplotypes with the B allele for SNP WUR10000125 and white bars represent haplotypes with the A allele for SNP WUR10000125.

Starting with haplotype 17 in Figure 3, haplotypes were grouped by phylogenetic distance based on two cuts in the phylogenetic tree to determine whether all B haplotypes carried the favorable causative variant. If an individual had at least one of the haplotypes to the right of the cut, the individual was placed in the group to the right of the cut, because of the identified dominance mode of action of the QTL [3]. To create grouping 1, the tree was cut to the left of haplotype 17B and to the left of 18B, to create the following haplotype groups: haplotype 17B vs. 15B plus 18B vs. all A haplotypes. Only one copy of haplotype 19B was present across trials and that animal’s other haplotype was 17B, so this animal was assigned to haplotype 17. Other groupings were created by moving the first cut up the tree, resulting in the groupings specified in Figure 4.Least squares means of the effect of each haplotype group for each grouping are in Figure 4. Results showed that all B haplotypes were associated with the favorable phenotype, and were significantly different from haplotypes that carried the A allele (p < 0.001) for both VL and WG42, except for grouping 2. For grouping 2, haplotype 18 had significantly lower VL than the other B haplotypes but was not significantly different from B or A haplotypes for WG. Haplotype 18 had a total of 21 copies (20 individuals) and was specific to trial 5; therefore, the results for group 2 may represent effects specific to this trial and this may confound the estimates of the effects of this haplotype. Haplotype 14, which was at the base of the A haplotypes (Figure 3), was not significantly different from the other A haplotypes (p > 0.83) and tended towards significance from the B haplotypes (grouping 4, p < 0.09) for VL. For WG42, haplotype 14 was not significantly different from the other B and A haplotypes. Haplotype 14 only had 11 copies and the SE associated with the effect of haplotype 14 were large. Moving further up the tree, the group with A haplotypes 14, 13, and 11 was not significantly different from the other A haplotypes but was significantly different from the B haplotypes for both VL and WG.In summary, the phylogenetic analysis segregated the haplotypes primarily by the allele at SNP WUR10000125, and there was a significant difference between the A versus B haplotype groups. Due to the high LD in the 487 kb region, relatively few haplotypes existed across the different populations for this region. This may indicate that there is little recombination in this region. In fact, across all ~1600 animals evaluated, only one recombination could be identified within the ~1 Mb region on SSC4. This recombination occurred between SNPs 31 and 32 in Figure 2, outside the 487 kb region. Therefore, this is a region with very little recombination, explaining the relatively few haplotypes that were present across all trials.

#### ***Additional regions associated with VL***

**Figure 5:**
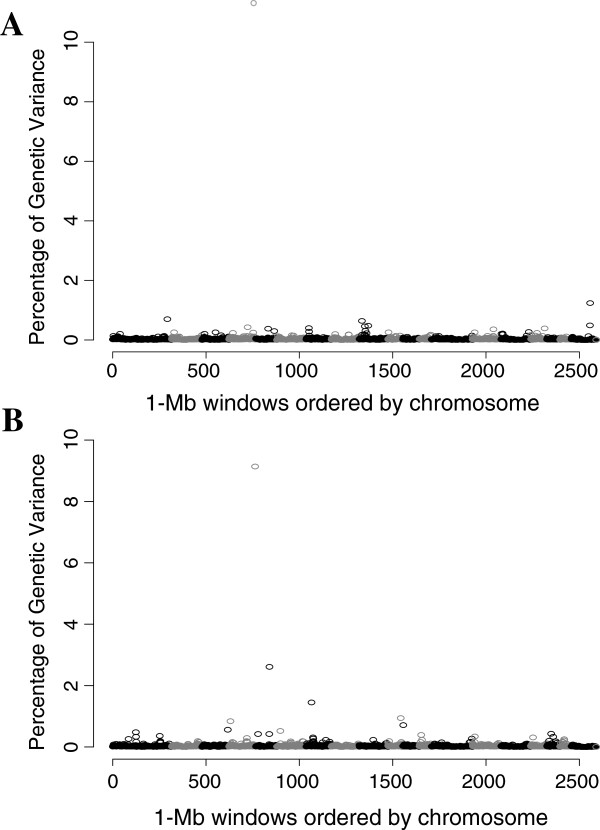
**Manhattan plot of the genome-wide association analysis of viral load (A) and weight gain to 42 days post infection (B).** Results are based on method Bayes-B with π = 0.99, and show the percentage of genetic variance that is explained be each non-overlapping 1 Mb window, labeled by index number of the first 1 Mb window and ordered by chromosome (1–18, X, and Y). Virus load was calculated as area under the curve of log-transformed viremia from 0 to 21 days post infection.

The results from the GWAS for VL are presented in Figure 5A. The top ten 1 Mb windows that explained the greatest percentage of genetic variance are in Table 6. The region on SSC4 explained the largest percentage of genetic variance at 13.2%. The remaining top 10 regions quickly dropped to less than 1% of the genetic variance and ranged from 1.24% to 0.39%. The second and third ranked regions will be further discussed.

**Table 6:** Top ten 1 Mb windows that explained the greatest percentage of genetic variance for virus load and weight gain

**Chromosome**	**Position**^**3**^	**Rank**	**Genetic variance (%)**
**Virus load**^**1**^			
4	139	1	13.2
X	113	2	1.24
1	292	3	0.70
9	13	4	0.64
X	112	5	0.49
9	47	6	0.48
9	29	7	0.46
4	99	8	0.43
7	13	9	0.40
16	74	10	0.39
**Total genetic variance**^**4**^		22.2	
**Weight gain**^**2**^			
4	139	1	9.14
5	72	2	2.61
7	27	3	1.45
10	67	4	0.94
4	7	5	0.84
11	1	6	0.71
3	138	7	0.56
6	18	8	0.52
1	123	9	0.48
17	22	10	0.43
**Total genetic variance**^**4**^		6.50	

^1^*VL* virus load calculated as area under the curve of log-transformed viremia between 0 and 21 days post infection (dpi); ^2^*WG* weight gain from 0 dpi to 42 dpi; ^3^Megabase position within a *Sus scrofa* chromosome; ^4^posterior mean of the total genetic variance from the genome-wide association analyses.

A region on SSCX, which included two consecutive 1 Mb regions, accounted for 1.73% of the genetic variance across all trials for VL but only 0.02% of the genetic variance for WG (Figure 5A, Table 6). There have been no reports of QTL associated with traits related to growth within 3 Mb up or down stream of this region (http://www.animalgenome.org/cgi-bin/QTLdb/SS/index). The region does include one reported QTL for blood pH in Meishan and Pietrain pigs infected with *Sarcocystis miescheriana*[13].

A region on SSC1 accounted for 0.70% of the genetic variance for VL across all trials and was the third highest region for VL (Figure 5A, Table 6). This region also explained only a small percentage of genetic variance for WG (0.03%). Three QTL associated with health traits have been reported for this region, including C3c concentration, alkaline phosphatase activity, and white blood cell counts (http://www.animalgenome.org/cgi-bin/QTLdb/SS/index). However, none of these QTL were identified under a PRRS challenge. Interesting candidate genes within 2 Mb on either side of this 1 Mb window include *DBC1* (*deleted in bladder cancer 1*) and *tumor necrosis factor* (*TNF*) *receptor-associated factor 1-like* (http://www.animalgenome.org). The *DBC1* gene is a tumor suppressor gene that is associated with programmed cell death [14]. The *TNF receptor-associated factor 1-like* gene is associated with antiviral activity [15].

#### ***Additional regions associated with WG***

The results from the GWAS for WG are presented in Figure 5B and the top 10 windows that explained the greatest percentage of genetic variance are in Table 6. Again, the region on SSC4 explained the largest percentage of genetic variance, at 9.1%. The percentage of genetic variance explained by the remaining regions ranged from 0.43% to 2.61%.

A region on SSC5 was the second highest and accounted for 2.6% of the genetic variance across all trials (Figure 5B, Table 6). This window does not appear to have an association with VL, as the percentage of genetic variance for VL was only 0.06%. Reports of QTL in this region associated with health in the pig include QTL for cholesterol level, haptoglobin concentration, alkaline phosphatase activity, interleukin-2 level, and interferon-gamma level (http://www.animalgenome.org/cgi-bin/QTLdb/SS/index). Interleukin-2 and interferon-gamma are both cytokines that respond to pathogen invasion of host cells. Interferon-gamma has been shown to inhibit PRRSV replication in macrophages [16,17]. There were two reports of QTL associated with average daily gain of healthy pigs that span the SSC5 region identified here (http://www.animalgenome.org/cgi-bin/QTLdb/SS/index).

A 1 Mb window on SSC7 (Mb 27) accounted for 1.45% of the genetic variance in WG across all trials (Figure 5B, Table 6) but only 0.06% of the genetic variance for VL. This region is within a ~5 Mb region (Mb 24 – 29) that contains several *Swine Leukocyte Antigen* (*SLA*) genes within the swine Major Histocompatibility Complex (http://www.ensembl.org, accessed March, 2013). To date, there have been 19 reports of QTL associated with production traits in this region, 18 of which were associated with BW or average daily gain from birth to market weight (pig genome assembly 10.2, animalgenome.org, accessed March, 2013). However, these QTL were identified using healthy, non-challenged pigs. The QTL identified here is for WG under PRRSV challenge and could be a new QTL; alternately one of the previously reported QTL could also be associated with WG under PRRSV challenge. Additional work is required to understand the underlying mechanism.

#### ***Cross-validation***

**Figure 6:**
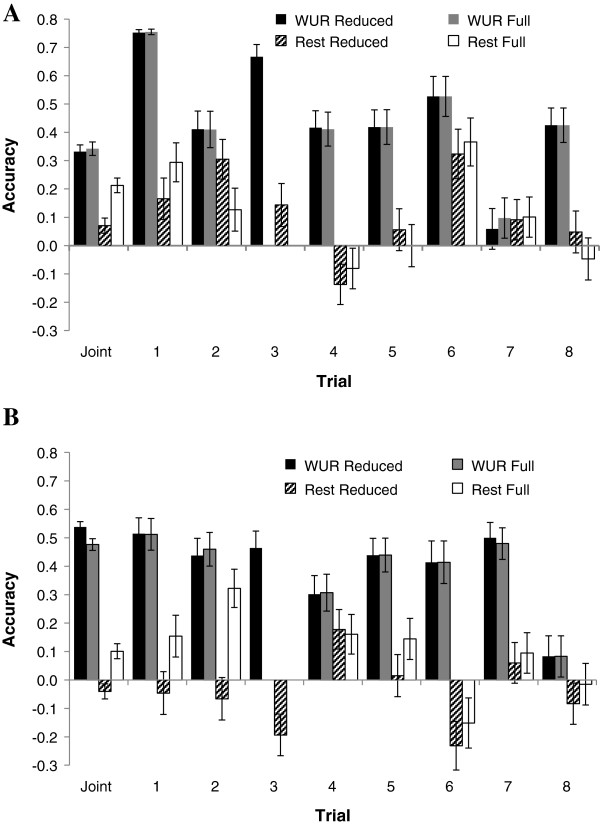
**Estimates of accuracy of genomic predictions in the validation population.** Accuracy based on the correlation between predictions and phenotypes adjusted for fixed effects, divided by the square root of heritability, for predictions obtained using reduced or full training populations (see Table 3). Predictions were based on the effect of SNP WUR10000125 (WUR) of *Sus scrofa* chromosome (SSC) 4 only, and based on the rest of the genome excluding the effect of the SSC4 region (Rest), for virus load (**A**, calculated as area under the curve of log-transformed viremia between 0 and 21 days post infection) and weight gain to 42 days post infection **(B)**.

Accuracies of predictions based on the SSC4 region and based on the whole genome including the SSC4 region, were previously reported by [3], using trials 1 through 3 for training and trials 4 and 5 for validation. On average, accuracies of GEBV based only on the SSC4 region were higher (0.24 for VL and 0.33 for WG) than accuracies of GEBV based on the whole genome, including SSC4 (0.20 and 0.31, respectively). Given those results, the hypothesis here was that the rest of the genome has much lower predictive ability than the region on SSC4.Accuracies of predictions based on SNP WUR10000125 and GEBV for the rest of the genome excluding the SSC4 region are shown in Figure 6A and B for VL and WG. Accuracies were, on average, much higher for SNP WUR10000125 than for the rest of the genome. Accuracies of predictions of genotypic values based on SNP WUR10000125 were very similar for the full and reduced training data but varied between validation trials from 0.08 to 0.51 for VL, with an accuracy of 0.48 and 0.54 when all trials were analyzed jointly, using the full and reduced training data, respectively, and from 0.06 to 0.75 for WG, with an accuracy of ~0.34 for the joint analyses.

For GEBV based on the rest of the genome and the reduced training data, accuracies centered around zero for VL, ranging from -0.23 to +0.18 for individual validation trials, with an accuracy of 0.10 and -0.04 for the joint analysis with the full and reduced training data, respectively. For WG, accuracies based on the rest of the genome tended to be positive, ranging from -0.14 to +0.31, with an accuracy of 0.21 and 0.07 for the joint analysis with the full and reduced training data. For the full training populations, accuracies increased substantially for trials 1 and 2, except for WG for trial 2, but had little impact on the other trials. These increases in accuracy for trials 1 and 2 were as expected because the full training data included data from the same crossbred genetics as used for validation.

Reasons for the generally observed poor predictive performance of GEBV across populations can be attributed to dominance, epistatis, and differences in linkage disequilibrium [18]. Accuracies of GEBV are generally lower when training and validation populations are more distantly related. For example, Saatchi et al. [19] partitioned data from Angus bulls into five groups using two methods; random allocation and k-means clustering, with the latter aiming at increasing relationships within groups and decreasing relationships between groups. On average, accuracies of GEBV when cross-validation in groups created by random allocation were higher than when groups were created by k-means clustering (0.65 vs 0.44). They concluded that this was due to closer relationships between training and validation populations when using random allocation. In the current study, the reduced training populations were not related to the validation populations. However, when validating on trials 1 and 2, trial 3 was included in the full training population, which resulted in some relatedness between training and validation populations. Inclusion of trial 3 in the training population resulted in increased accuracies for trials 1 and 2 compared to the absence of trial 3 in the reduced training population, which agrees with the results of Saatchi et al. [19]. However, SE were large for all estimates of accuracy. Furthermore, the increase in relationships between training and validation populations was confounded with the increased size of the training population.

## Conclusions

Traits associated with PRRS following experimental infection in growing pigs appear to have a strong genetic component, with moderate estimates of heritability for VL and WG, along with a large QTL on SSC4. Strong LD is present in the SSC4 region. However, combining all eight trials broke some of the LD in the ~1 Mb region, resulting in a smaller haplotype. This reduced haplotype block could be useful to identify the causative mutation. A phylogenetic analysis separated the haplotypes by the allele at the SNP that explains nearly all the genetic variance for VL and WG that is contributed by the region, providing additional evidence that SNP WUR10000125 is the most informative SNP in the region (along with three other SNPs that were in complete LD with the WUR SNP) and is likely in very high LD with the causative mutation. The most common B haplotype was present in all breeds and lines represented in this study and was likely present before the segregation of the breeds. Cross-validation for the SSC4 QTL resulted in high estimates of accuracy when validating across populations for VL and WG, which can benefit selection strategies without infecting piglets with PRRSV. After accounting for the SSC4 region, the rest of the genome had little predictive ability for VL and WG across unrelated populations. Additional work is required to determine predictive ability within a population. With the effect of the SSC4 region present in all breeds and lines analyzed here, there is a good possibility that the effect is present in more populations and that genetic progress for PRRS tolerance or resistance can be made. However, additional work is needed to verify the effects of this region on challenges with different PRRSV strains and in the field.

Other genomic regions associated with VL and WG were identified also but with small effects. In general, these regions will probably not play a major role in reducing the economic impact of PRRS.

The genomic region identified in this study can be used for marker-assisted selection for response to PRRS in growing pigs. The implementation of selection for these markers will not make pigs completely resistant to PRRS but will increase the well-being of the animals and reduce the financial impact of PRRSV infections by limiting reductions in growth rate and other negative effects of PRRS following infection. Further work is required to investigate the effects of these QTL against different PRRSV strains and in the field where pigs are subjected to many additional environmental stressors.

## Competing interests

The authors declare that they have no competing interests.

## Authors’ contributions

NJB conducted the statistical analyses, interpretation of results, and wrote the manuscript, AB conducted the initial haplotype analysis studies, RRRR conceived the study and led the animal infection trials and sample collection, JKL conceived the study and coordinated the handling, storage, and sample preparation for DNA, JMR helped conceive the study, coordinated data base development and helped with interpretation of analyses, JCMD coordinated and oversaw statistical analysis of the data and contributed to interpretation of results and writing the manuscript. All co-authors reviewed and contributed to development of the manuscript. All authors read and approved the final manuscript.
